# Early activation of bioenergetic metabolism powers bacterial spore germination

**DOI:** 10.1073/pnas.2510996122

**Published:** 2025-12-24

**Authors:** Pooja Gupta, Rebecca Caldbeck, Rowan C. Walters, Elodie C. Wells, Bethany L. Hardman, Graham Christie, Roger J. Springett, James N. Blaza

**Affiliations:** ^a^York Structural Biology Laboratory, Department of Chemistry, University of York, York YO10 5DD, United Kingdom; ^b^York Biomedical Research Institute, University of York, York YO10 5DD, United Kingdom; ^c^Department of Chemical Engineering and Biotechnology, University of Cambridge, Cambridge CB3 0AS, United Kingdom; ^d^CellSpex Ltd., Northampton NN6 9HF, United Kingdom

**Keywords:** spore, germination, bioenergetics, cytochrome, spectroscopy

## Abstract

Dormant bacterial spores germinate to become vegetative cells upon germinant exposure. Despite many germinants being energy sources, bioenergetic processes have been overlooked as germination can proceed, albeit slowly, without exogenous energy sources. Here, we apply remission spectroscopy to noninvasively measure energization of the electron transport chain (ETC) in germinating spores. In *Bacillus megaterium* and *Bacillus subtilis*, energization of cytoplasmic metabolism and the ETC occurs early in germination, before or alongside water ingress and bulk CaDPA efflux. The *aa*_3_-type oxidases (Qox, Cta) accumulate nonradical ferryl intermediates of their catalytic cycle, slowed by a high membrane potential. The Yth isoform of the *bd* oxidase, present in spores, allows rapid electron transfer to O_2_ when the *aa*_3_-type oxidases are hindered, establishing a role for this enzyme. Deletion of Yth slows the initiation of “absorbance”/attenuance loss, directly linking bioenergetic processes to germination. We propose a powered germination model, where the Ger-mediated signaling cascade and bioenergetic processes occur in parallel and are mutually influenced by each other. This model explains why germination on energy-rich molecules (e.g., glucose) is often much faster than on energy-poor ones (e.g., KBr).

## Significance

Bacterial spores can survive, dormant, for thousands of years and yet are able to germinate into vegetative cells in about an hour. The current consensus is that resumption of metabolism is a late event in germination occurring only once the spores have sensed their germinants and rehydrated. Here, a biophysical approach called remission spectroscopy is used that measures haem groups within energy-transducing cytochrome enzymes of intact, germinating spores. We find that energy metabolism actually starts right at the beginning of the germination process. Our observations upturn the view that germination is only a passive process of solutes flowing down concentration gradients. Instead, we see active energization of the metabolic system that can be powering processes such as transport or macromolecular synthesis.

Bacteria of the phylum Firmicutes sporulate as a survival strategy: Once matured, they maintain viability for prolonged periods of time, allowing them to endure unfavorable conditions (1). When conducive growth conditions return, spores sense “germinants” such as sugars and amino acids, and even nonnutrient signals (e.g., K^+^) through their germinant (Ger) receptors to initiate germination (2). Remarkably, through germination they transition from being a dormant body to a metabolically active cell in about an hour (3).

Mature *Bacillus* spores have an onion-like multilayered architecture that underpins their exceptional robustness (4). The DNA-containing core contains precipitated calcium-dipicolinic acid chelate (CaDPA) at >800 mM (5), and has a gel-like consistency with little mobile water (6, 7). Around the core is a cytoplasmic membrane, which harbors the Ger receptors and SpoVA channel proteins that are directly involved in the initiation of germination (8, 9), along with respiratory enzymes of the electron transport chain (ETC) (10–12). The cytoplasmic membrane presents a permeability barrier, where the constituent lipids are mostly immobilized (13). Around the cytoplasmic membrane is the germ cell wall, peptidoglycan-based cortex, outer membrane, proteinaceous coat, and an exosporium layer in some species. Germination has been described in two stages (14, 15). In stage I, germinant recognition leads to substantial efflux of H^+^, K^+^, Na^+^ (16–18) out of the spore along with slow leakage of CaDPA (19). As more CaDPA from the spore core is released and replaced by water (rehydration), the spore moves into stage II, where activation of cortex-lytic enzymes allows spore swelling, emergence, and elongation, followed by the first cell division (20, 21). These later processes constitute outgrowth. Core rehydration progresses during both stages, visualized as phase transition taking between 2 and 3 min for a spore (19, 22).

Cells maintain certain reactions away from equilibrium to create energy-carrying chemical potentials. These reactions are coupled to energetically unfavorable reactions to drive them. The most well-known of these is the phosphorylation of ADP to make ATP by ATP synthase, driven by the imbalance of charge (ΔΨ) and pH (ΔpH) established across an energy-transducing membrane. This imbalance is created by the enzymes of the respiratory chain pumping protons as they pass electrons from low-potential donors (e.g., NADH) to high potential acceptors (e.g., O_2_). In dormant spores, the adenine nucleotide pool is found largely as ADP/AMP and the NAD(P)H pool is oxidized, so they are incapable of powering reactions (23–25) and dormant spores are therefore considered energetically depleted. Observations that these pools are not replenished before germination begins and that hydration can occur without O_2_ have led to the consensus view that oxidative energy metabolism is not a prerequisite for initiation of germination, and restarts much later (4, 14, 23, 26–28). Interestingly, like ATP generation, establishment of ΔΨ is a fundamental mechanism of energy conservation and a plausible energy source for germinating spores. Single spore measurements have suggested that ΔΨ exists before germination, although this is most likely an artifact because thioflavin-T binds nonspecifically to the spore coat (29, 30).

Bioenergetic reactions are fast, so measurements requiring cellular disruption are compromised as the in vivo state is altered during sample preparation. This means effective tools for measuring bioenergetic reactions in vivo have historically been lacking. For bacterial spores where disruption is nontrivial, the dearth of suitable techniques has led to bioenergetic metabolism being overlooked in favor of other processes in the germination cascade that are easier to measure. Here, we use a bioenergetic chamber to make visible-wavelength spectroscopic and O_2_ consumption measurements on intact spores germinating on their favored germinants: *Bacillus megaterium* on glucose; *Bacillus subtilis* on alanine and glucose. We measure glucose oxidation and ETC reduction before detectable germination in *B. megaterium*; in *B. subtilis*, detectable germination and glucose-powered ETC reduction start simultaneously. These observations challenge models of germination in which metabolism restarts only once core rehydration is complete. We propose a powered germination model in which germinating spores are respiring and energized.

## Results

### A Bioenergetic Chamber Measures Electron Transfer to Cytochromes and O_2_ During Spore Germination.

**Fig. 1.**
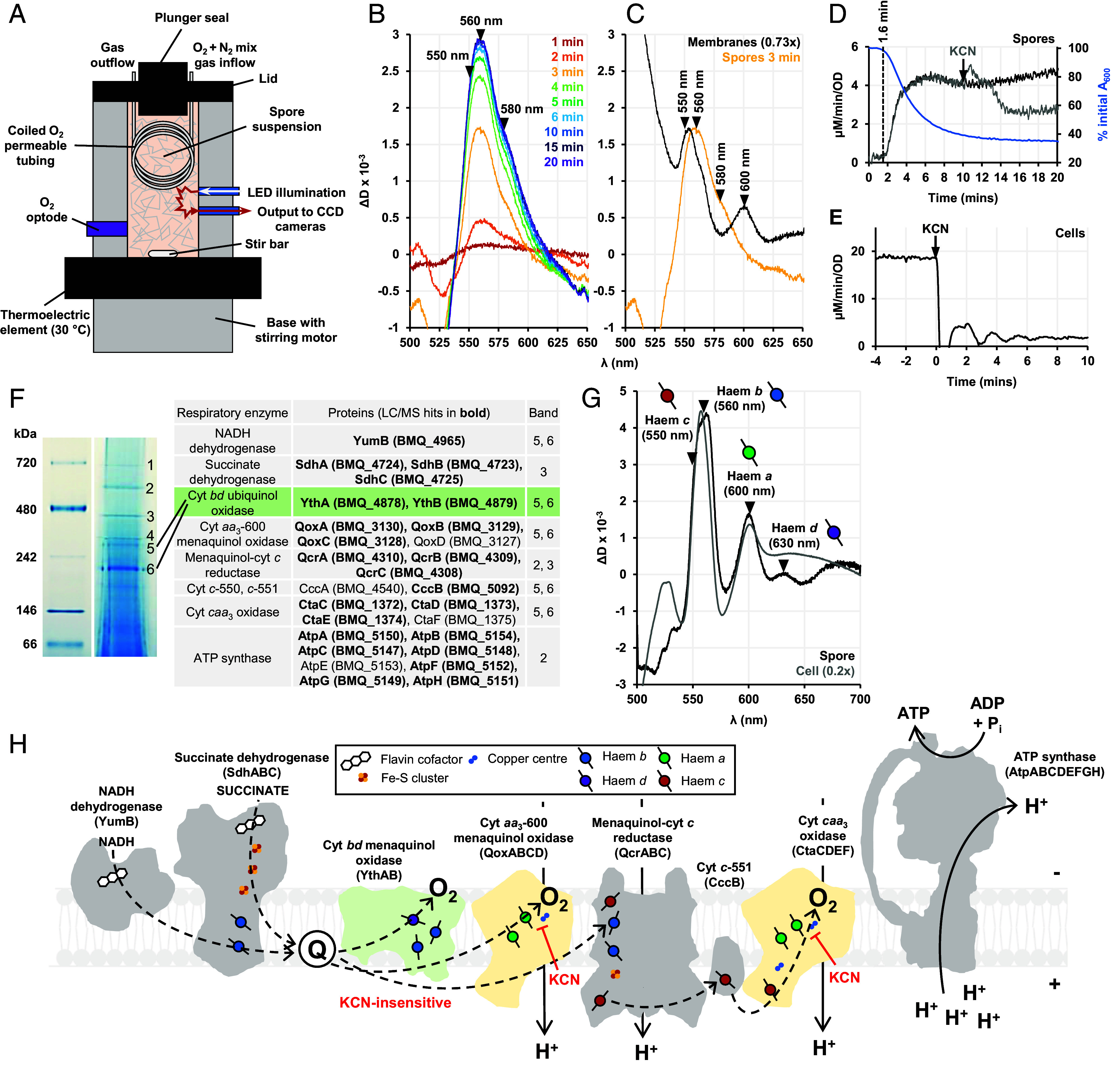
Remission haem spectroscopy on germinating *B. megaterium* spores. (*A*) Schematic of the bioenergetic chamber where a dense suspension of heat-shocked spores is maintained at 30 °C, 120 µM O_2_ and germination is initiated at t = 0 min with the addition of 10 mM glucose; spectra are recorded by the CCD-spectrograph system. (*B*) Difference spectra generated for the indicated time points after glucose addition at t = 0 min. (*C*) The 3 min spectrum of germinating spores from (*B*) compared with a spectrum of isolated spore membranes after 3 min of NADH reduction. (*D*) OCR of spores germinating with 10 mM glucose (black), poisoned with 1 mM KCN (gray), along with the germination curve (blue). (*E*) OCR of late-exponential *B. megaterium* cells inhibited by 1 mM KCN. (*F*) BN-PAGE gel of DDM-solubilized spore membranes and bands labeled 1 to 6 subjected to LC/MS analysis. The table lists the proteins of respiratory complexes (with gene IDs) found to be the most abundant in these bands. The cytochrome *bd* spore isoform YthAB is highlighted (green). (*G*) The sodium dithionite-reduced minus air-oxidized spectra of isolated cell and spore membranes. (*H*) Scheme of the ETC present in dormant spores based on the LC/MS analysis of BN-PAGE gel bands in (*F*). The CN^−^-sensitive *aa*_3_-type oxidases (Qox and Cta) are shown in yellow and the CN^−^-insensitive *bd* oxidase (Yth) in green.

Haem groups present in respiratory cytochromes have characteristic visible-wavelength absorbance bands that change as they undergo reduction. These changes were used as spectroscopic handles in our “bioenergetic chamber” (Fig. 1*A*) for remission spectroscopy where instead of transmitted light, light backscattered (“re-emitted”) off the turbid suspension is used (31). An O_2_ optode simultaneously measured O_2_ consumption rate (OCR) and the samples were maintained at a constant O_2_ concentration by a gas delivery system (32).

**Fig. 2.**
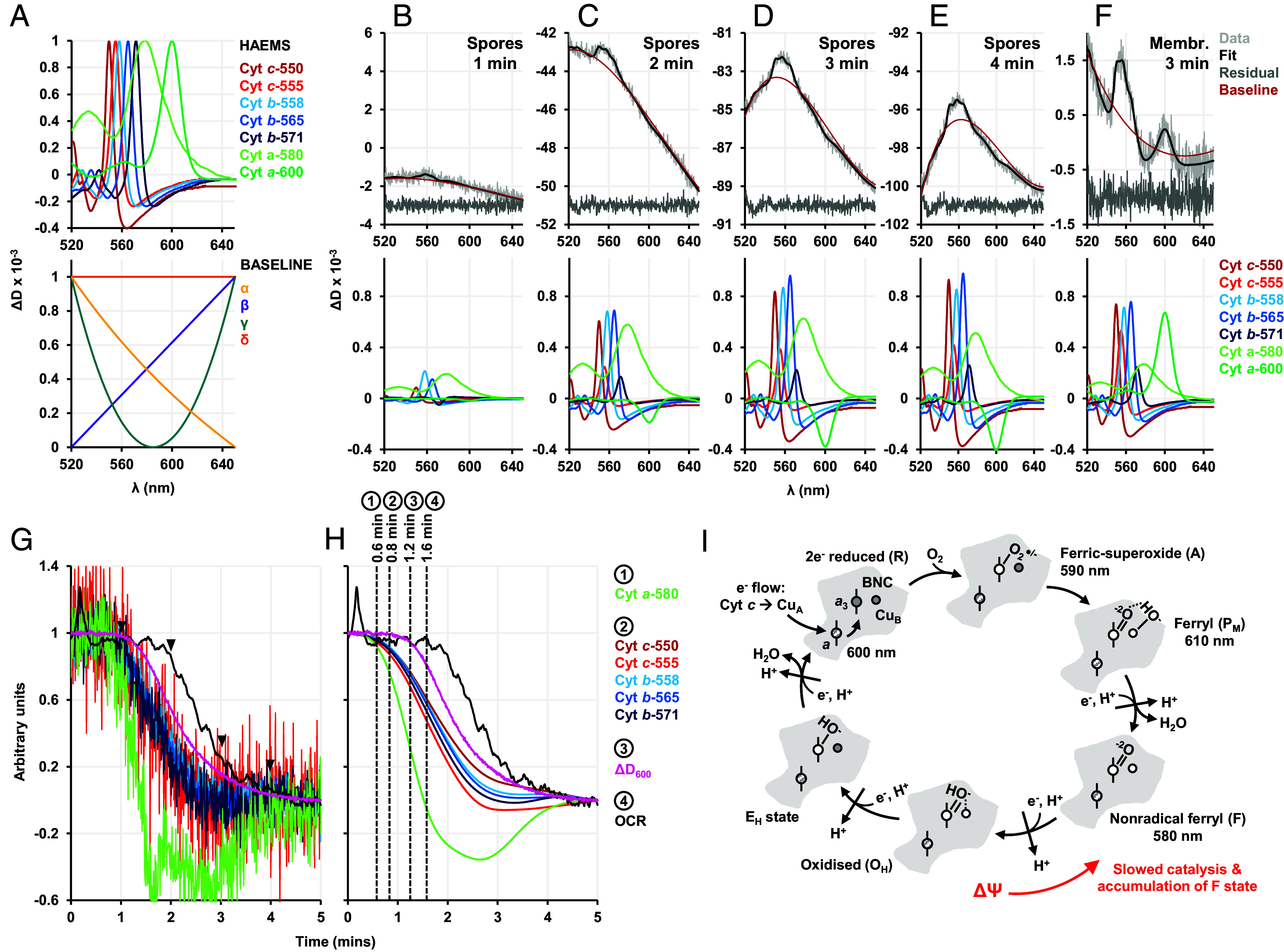
Spectral unmixing and the kinetics of spectral changes in germinating spores. (*A*) The haem (*Top*) and baseline (*Bottom*) components of the fitting template. (*B*–*E*) are spectra of spores germinated with 10 mM glucose, (*F*) is a spectrum of isolated spore membranes that were reduced with 1 mM NADH. (*B*–*F*) *Top* panel shows the raw data, the fit imposed, the residuals (suitably offset from 0 for clarity), and the sum of the fitted baseline components. The *Bottom* panel shows the fitting of the haem components. (*G*) Normalized kinetic traces (fitting vs. time) of selected haem components, change in attenuance (ΔD_600_), and OCR for spores germinated with 10 mM glucose. Arrowheads indicate the time points 1, 2, 3, and 4 min for which the decomposition analysis is demonstrated in (*B*–*E*). (*H*) shows the Savitzky–Golay smoothed haem traces along with the unsmoothed germination signal and OCR traces. Events in clusters 1, 2, 3, and 4 start 0.6, 0.8, 1.2, and 1.6 min, respectively. (*I*) The canonical catalytic cycle of *aa*_3_-type oxidases showing the catalytic intermediates formed at the binuclear center (BNC, composed of Cu_B_ and haem a_3_) and the characteristic absorbances of their peaks. Gray color of the haem/copper group indicates reduced Fe^2+^/Cu^+^, white color indicates oxidized Fe^3+^/Cu^2+^ state. Haem *a* can be oxidized/reduced, shown as a shaded haem center. For every electron and proton transferred to the BNC, another proton is pumped as indicated by the arrows pointing outward. The text and arrow in red show the effect of ΔΨ on the catalytic cycle.

Glucose was added to the suspension of dormant *B. megaterium* spores in the bioenergetic chamber. Spectral changes were observed arising from redox changes in the abundant ETC cytochromes (Fig. 1*B*). Overlapping peaks at 550 nm and 560 nm indicated reduction of haem *c* and *b* groups, respectively. Intriguingly, despite the presence of two *aa*_3_-type oxidases (Qox and Cta) in the ETC (Fig. 1*H*), the characteristic signal of haems *a* (peak at 600 nm) was not observed, instead a shoulder was seen at 580 nm. The 580 nm signal is associated with the ferryl intermediate “F” of the *a*_3_-haem in the catalytic cycle of *aa*_3_-type oxidases (Fig. 2*I*). This is a minor intermediate under turnover conditions in purified enzyme preparations (33–35), has been observed in isolated mitochondria and submitochondrial particles (36–39), but this is the first report of the F intermediate being sufficiently abundant to be detectable in vivo. Under physiological conditions in mammalian cells, the ferryl intermediates are not detectable, and reduced haem *a* is the dominant spectral signature of cyt *c* oxidase activity (40). We isolated spore membranes, reduced them with NADH and found the haem *a* peak at 600 nm (Fig. 1*C*). This showed that Cta and Qox in spores are indeed canonical *aa*_3_-type oxidases but something during germination places them in a different catalytic state compared to vegetative cells.

### The Spore ETC Is Proteomically and Functionally Distinct from that of a Vegetative Cell.

Given the consensus that bioenergetic processes start later in germination, we were surprised to measure germinating spores consuming O_2_ concomitantly with the loss of absorbance at 600 nm (Fig. 1*D*). To investigate the nature of this O_2_ consumption, we added CN^-^, a tight-binding inhibitor of *aa*_3_-type oxidases. We found that unlike in vegetative cells (Fig. 1 *D* and *E*), O_2_ consumption in spores was CN^-^-resistant, consistent with previous studies (41). There is spectroscopic evidence for the presence of an alternative CN^-^-insensitive oxidase in spores: a cytochrome *bd* oxidase (42). We confirmed this by isolating membranes from *B. megaterium* spores and vegetative cells and measuring the difference spectra; the 630 nm peak in the spore membrane oxidized-reduced difference spectrum is diagnostic for haem *d* (Fig. 1*G*). As cytochrome *bd* enzymes are high affinity terminal oxidases typically used under microaerobic/stress conditions (43), we were surprised to find it in the spores of *B. megaterium*, an aerobic species cultured in O_2_-replete conditions. The genome of both *B. megaterium* and *B. subtilis* encodes two *bd* oxidase enzymes, CydAB and YthAB (44). DDM-solubilized *B. megaterium* membranes were resolved on a BN-PAGE gel and mass spectrometry on these bands revealed only peptides for YthAB (Fig. 1*F* and Dataset S1), matching studies of other *Bacillus* spore membrane proteomes (10, 45). During aerobic exponential growth of *B. subtilis*, YthAB was absent and a physiological role for it is not yet identified (44, 46).

In addition to our observation of Yth in spores, we could only detect the YumB isoform of the NADH dehydrogenase-quinone reductase, rather than enzymes encoded either by *ndh* or *yutJ*. We found proteomic evidence for most of the canonical respiratory enzymes that lack isoforms, which are listed and illustrated in Fig. 1 *F* and *H*, respectively, suggesting that the use of YumB and YthAB attunes the spore bioenergetic system for germination.

### Spectral Unmixing Reveals Electron Transfer Starts Before Detectable Hydration in Germinating Spores.

To understand the kinetics of electron transfer and hydration, we used decomposition to unmix the observed remission spectra into the additive haem and baseline components (Fig. 2*A*). This analysis enabled visualization of how individual spectral components changed with respect to each other over time (Fig. 2 *B*–*F*). A smooth baseline was included to account for the large scattering changes that occurs as the spores germinated. We also directly plotted the loss of attenuance at 600 nm (ΔD_600_), which reflects the same physical phenomenon as loss of “absorbance,” widely used as the “germination” signal. It is caused by bulk CaDPA efflux and core hydration bringing the refractive index of germinating spores closer to that of the surrounding medium.

In the kinetic traces generated through decomposition (Fig. 2 *G* and *H*), we distinguished four clusters of events, designated C1 to C4. Interestingly, reduction of *b* and *c* cytochromes (C2) started almost half a minute before ΔD_600_ (C3) and a minute before change in the OCR trace (C4), indicating that electrons are entering the ETC before detectable germination but are not traveling all the way down to O_2_. Furthermore, the *aa*_3_-type oxidase F intermediate (C1) accumulated faster than the reduction of *b* and *c* haem groups, attributable to the reduction of Qox, which is reduced directly from quinol in the Q-pool.

It was surprising that the reduction of cytochromes was not immediately followed by an increase in OCR as electrons are normally transferred through ETCs in under a second (47, 48). To rationalize this observation, we examined the extensively studied catalytic cycle of *aa*_3_-type oxidases (Fig. 2*I*) (35). With the delivery of 4 protons and 4 electrons to the BNC, one O_2_ is fully reduced to two H_2_O molecules. Each proton and electron transfer step to the BNC is coupled with the pumping of an additional proton through the membrane, building ΔΨ and the proton-motive force (PMF). A high PMF pushes against the catalytic cycle, and the F intermediate accumulates (36), which is unable to react with O_2_. The earliest recorded event in the *B. megaterium* germination cascade is the release of cations (18, 49) but whether this release builds ΔΨ depends on the countermovement of charge. Detection of the F intermediate is indirect evidence that a substantial ΔΨ is established at the early stages of germination. This could explain the presence of the cytochrome *bd* Yth: as it lacks vectorial proton pumping, it will not be slowed by a high PMF to the same extent, allowing rapid transfer of electrons to O_2_ as spores germinate.

### Glucose-Powered Metabolism Starts Early in Germination.

Incompatible with the current consensus, our data showed some ETC filling before detectable germination and O_2_ consumption starting around the time of germination. Therefore, we sought to reevaluate the timeline of metabolic events in the germination cascade.

**Fig. 3.**
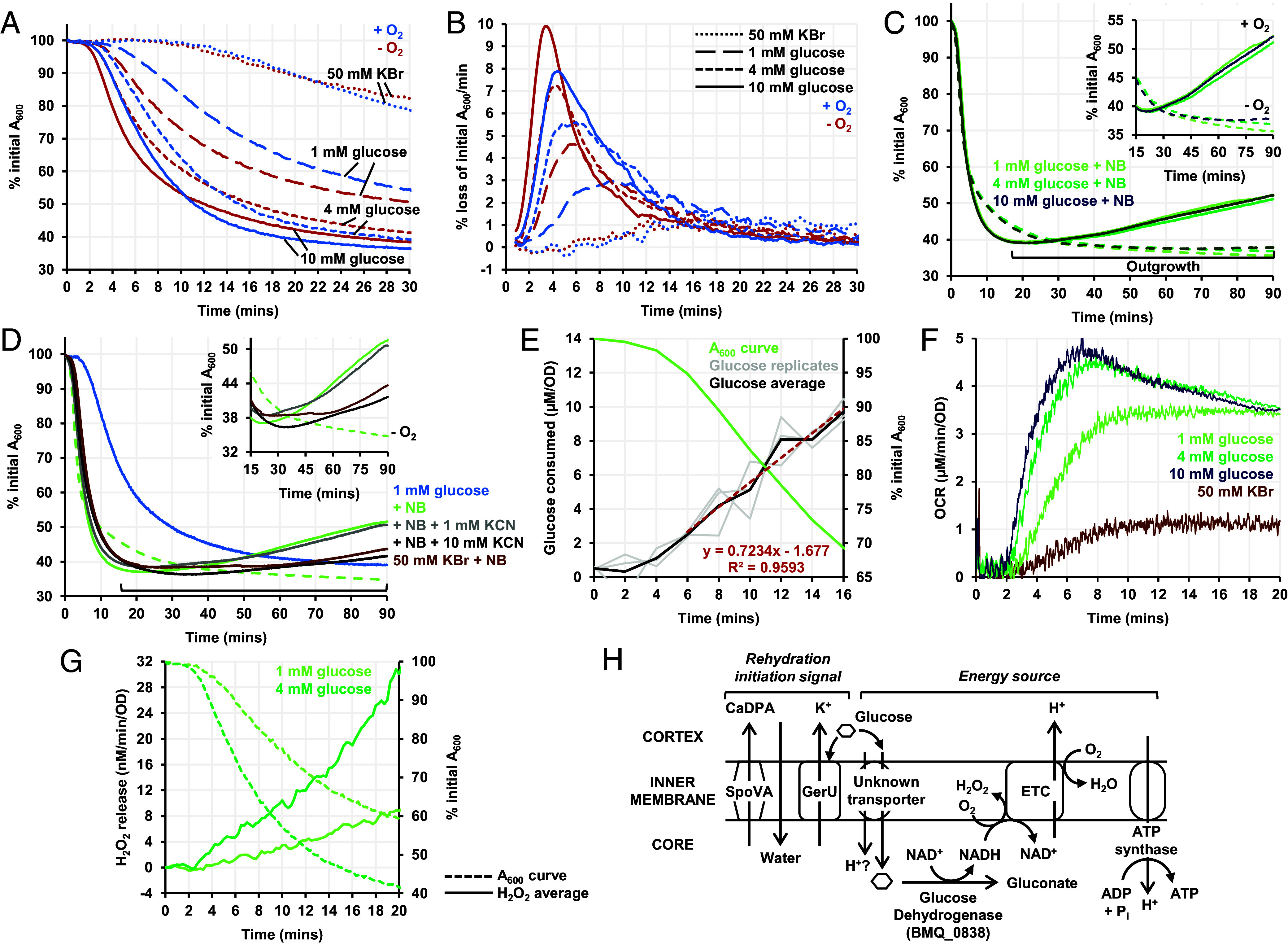
Role of glucose as a germinant and energy source. (*A*) Germination curves (expressed as % initial absorbance at 600 nm vs. time) with 50 mM KBr (small dots), 1 mM glucose (long dashes), 4 mM glucose (short dashes) and 10 mM glucose (solid line), under oxic (blue) and anoxic (red) conditions. (*B*) Rate of spore germination (% loss of initial absorbance/min) with 50 mM KBr, 1 mM glucose, 4 mM glucose, and 10 mM glucose under oxic and anoxic conditions. (*C*) Outgrowth curves for spores germinated with nutrient broth and 1 mM glucose (light green), 4 mM glucose (dark green) and 10 mM glucose (teal), under oxic (solid line) and anoxic (dashed line) conditions. (*D*) Outgrowth curves for spores germinated with 1 mM glucose alone (light blue), 1 mM glucose and nutrient broth (light green), 1 mM glucose and nutrient broth under anaerobiosis (light green dashed), 1 mM glucose, nutrient broth, and either 1 mM KCN (gray) or 10 mM KCN (black). (*E*) Average glucose consumption by spores germinated with 1 mM glucose (black) across replicates (gray) along with the germination curve obtained with 1 mM glucose (green). Glucose was consumed at a rate of 0.72 μM/min (normalized by OD) in the 6- to 16-min period. (*F*) OCRs measured without the O_2_ delivery system, for germinating spores germinated with 1 mM glucose (light green), 4 mM glucose (dark green) and 10 mM glucose (dark blue), and 50 mM KBr (maroon). (*G*) Rate of H_2_O_2_ release by spores germinated with 1 mM glucose (light green) and 4 mM glucose (dark green). (H) Model showing glucose linking germination with bioenergetics in *B. megaterium*.

We used different concentrations of glucose to germinate spores in the presence and absence of O_2_, using traditional loss of absorbance measurements. As previously observed (50), the extent and rate of absorbance/attenuance loss increased with glucose concentration (Fig. 3 *A* and *B*). Unexpectedly, the absence of O_2_ increased the rate of germination; a likely explanation is that oxidative metabolism could not occur so more glucose was available to activate GerU. In support of this hypothesis, germination with KBr, a nonmetabolizable germinant, was unaffected by the absence of O_2_. Outgrowth relied on the presence of both O_2_ and a complete medium and was inhibited by ~60% with excess KCN (Fig. 3 *C* and *D*).

Previous studies came to contradictory conclusions about the timeline of glucose uptake and consumption during germination (49, 51). In our experimental system, glucose concentration in the medium started decreasing (Fig. 3*E*) alongside an increase in OCR that was glucose concentration-dependent (Fig. 3*F*), both concomitantly with germination. Reactive O_2_ species (ROS) generation is an important signature of oxidative metabolism and H_2_O_2_ production was also measured at this time, proportional to glucose concentration (Fig. 3*G*).

In *B. megaterium*, glucose can be catabolized by the glycolytic, pentose-phosphate and gluconate pathways (52). All three pathways become operational in germinating spores, but the gluconate pathway is responsible for more than half of the glucose catabolism in the first 15 min, generating NADH (53). BMQ_0838 was found to encode the spore isoform of glucose dehydrogenase (Gdh), the first enzyme in the gluconate pathway (*SI Appendix*, Fig. S1 and Table S2 and Dataset S1). The dominance of the gluconate pathway implies that the majority of glucose taken up is unphosphorylated, mediated by a nonphosphotransferase system permease. A potential candidate is GlcU where transport is PMF-driven (54). *GlcU* and the spore-specific *gdh* are in the same operon in *B. subtilis* and *B. megaterium*; GlcU has also been identified in the *B. subtilis* spore membrane proteome (45).

Finally, our model for glucose-powered oxidative metabolism during germination (Fig. 3*H*) relies on the NADH produced being able to rapidly reduce the ETC. Kinetic assays showed that spore membranes oxidized NADH nearly twice as fast as membranes from vegetative cells, and were also more CN^−^-resistant due to Yth function (*SI Appendix*, Table S1).

### The *ythAB*-Encoded Cytochrome *bd* Oxidase Drives ETC Function in Germinating Spores.

We have established that the spore ETC is rapidly reduced early in germination, powered by glucose metabolism. We now turn to the role of the spore-specific *bd* oxidase, Yth. Given that Qox and Cta were operating in distinct and slower catalytic regimes compared to vegetative cells, we speculated that Yth could be acting to relieve reductive pressure.

**Fig. 4.**
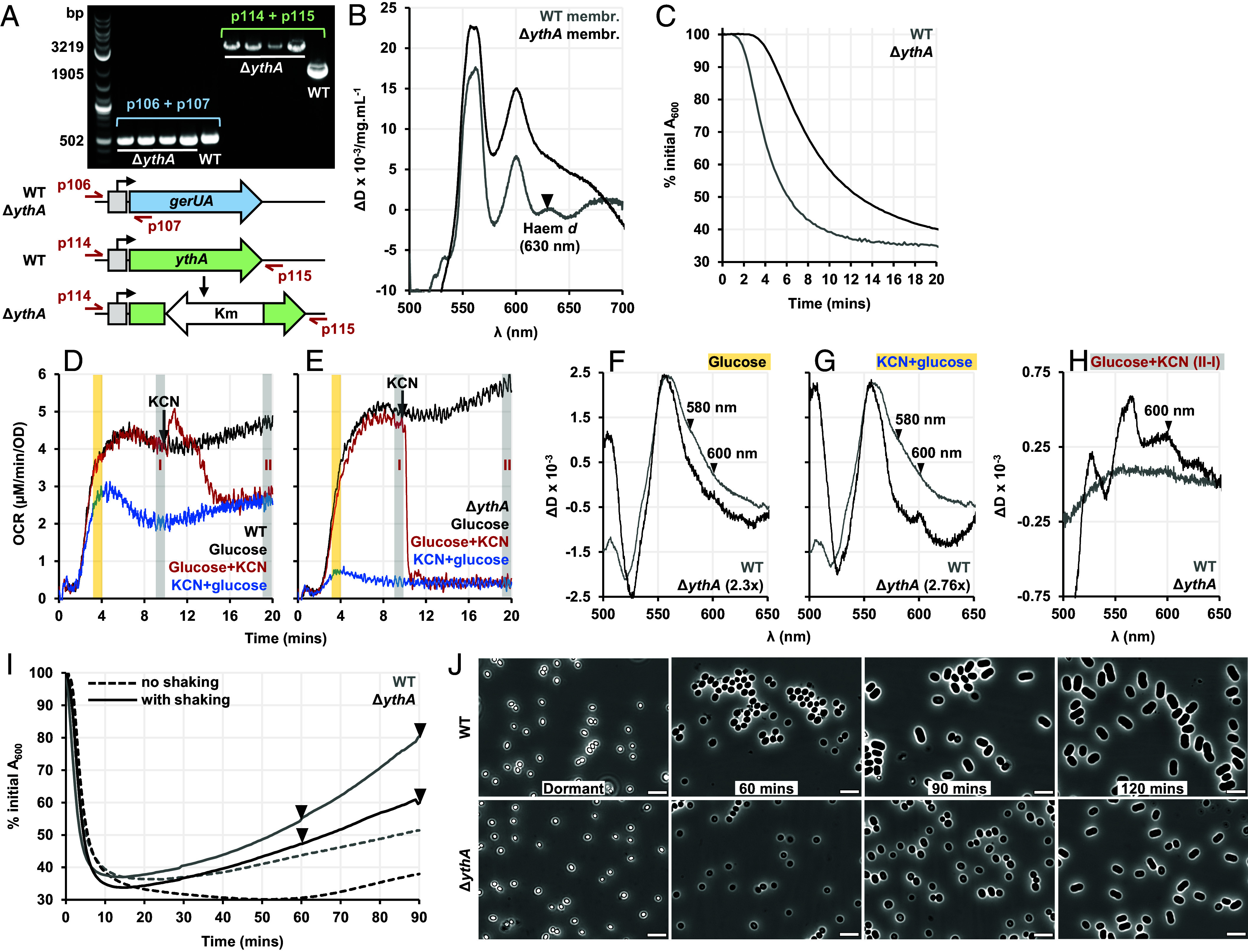
Effect of *ythA* deletion on *B. megaterium* spore germination. (*A*) The WT and Δ*ythA* genotypes confirmed by PCR. Both strains have the plasmid-borne glucose receptor GerU (*gerUA* tested with primers p106 and p107) but Δ*ythA* is an insertional deletion mutant of *ythA* (tested with primers p114 and p115). (*B*) The dithionite-reduced minus air-oxidized spectra of isolated membranes from dormant WT (gray) and Δ*ythA* (black) spores. (*C*) Germination curves obtained for the WT (gray) and Δ*ythA* (black) spores with 10 mM glucose. (*D* and *E*) OCRs measured for WT and Δ*ythA* spores germinated with 10 mM glucose (black), when 1 mM KCN was added either before glucose (blue) or 10 min after glucose (red). (*F* and *G*) Difference spectra generated for WT (gray) and Δ*ythA* (black) spores. (F and G) show the 4-min spectra indicated by the yellow shaded region in (*D* and *E*), with glucose alone (*F*) or in the presence of KCN (*G*). (*H*) shows the difference spectra (II–I), indicated by gray shaded regions in (*D*) and (*E*), when KCN was added 10 min after glucose. (*I*) Outgrowth curves for WT (gray) and Δ*ythA* (black) spores germinated with 10 mM glucose and nutrient broth, when the 96-well plate was shaken (solid line) or not (dashed line). (*J*) Phase-contrast micrographs of WT (*Top*) and Δ*ythA* (*Bottom*) spores taken at the time points indicated by arrowheads during the outgrowth experiment (with shaking) shown in (*I*). (Scale bar, 5 μm.)

YthA, the haem-containing catalytic subunit of the heterodimeric cytochrome *bd* oxidase, was deleted in *B. megaterium* spores (Fig. 4*A*). Deletion of *ythA* was confirmed by remission spectroscopy on isolated spore membranes (Fig. 4*B*). Compared to WT, Δ*ythA* spores were slower to initiate absorbance/attenuance loss with 10 mM glucose (Fig. 4*C*) but achieved a comparable OCR (Fig. 4*D*). However, unlike in WT spores, O_2_ consumption in germinating Δ*ythA* spores was fully abolished by 1 mM KCN (Fig. 4 *D* and *E*), consistent with the deletion of a CN^-^-resistant *bd* oxidase without induction of Cyd, the other *bd* oxidase in the genome. Difference spectra in Fig. 4 *F*–*H* show that compared to WT, Δ*ythA* spores had less of the 580 nm F species, and the small spectral peak at 600 nm became stronger when KCN inhibited O_2_ consumption and reduced the haem *a* centers further (this peak is distinct from the much larger ΔD_600_ signal stemming from changes in background scattering). This change suggests that in the absence of Yth, the *aa*_3_-type oxidases were pushed to operate faster and some of the 600 nm intermediate accumulated, as it did in vegetative cells.

WT and *ΔythA* spores were germinated in a 96-well plate (Fig. 4*I*) and imaged with phase-contrast microscopy (Fig. 4*J*). For *ΔythA* spores, outgrowth was much slower when the 96-well plate was not shaken, as the oxygenation was insufficient and the high-affinity cytochrome *bd* oxidase was absent. With shaking, both strains could outgrow better but *ΔythA* spores still lagged behind WT.

### Demonstrating that Similar Principles Underlie *Bacillus subtilis* Spore Germination.

**Fig. 5.**
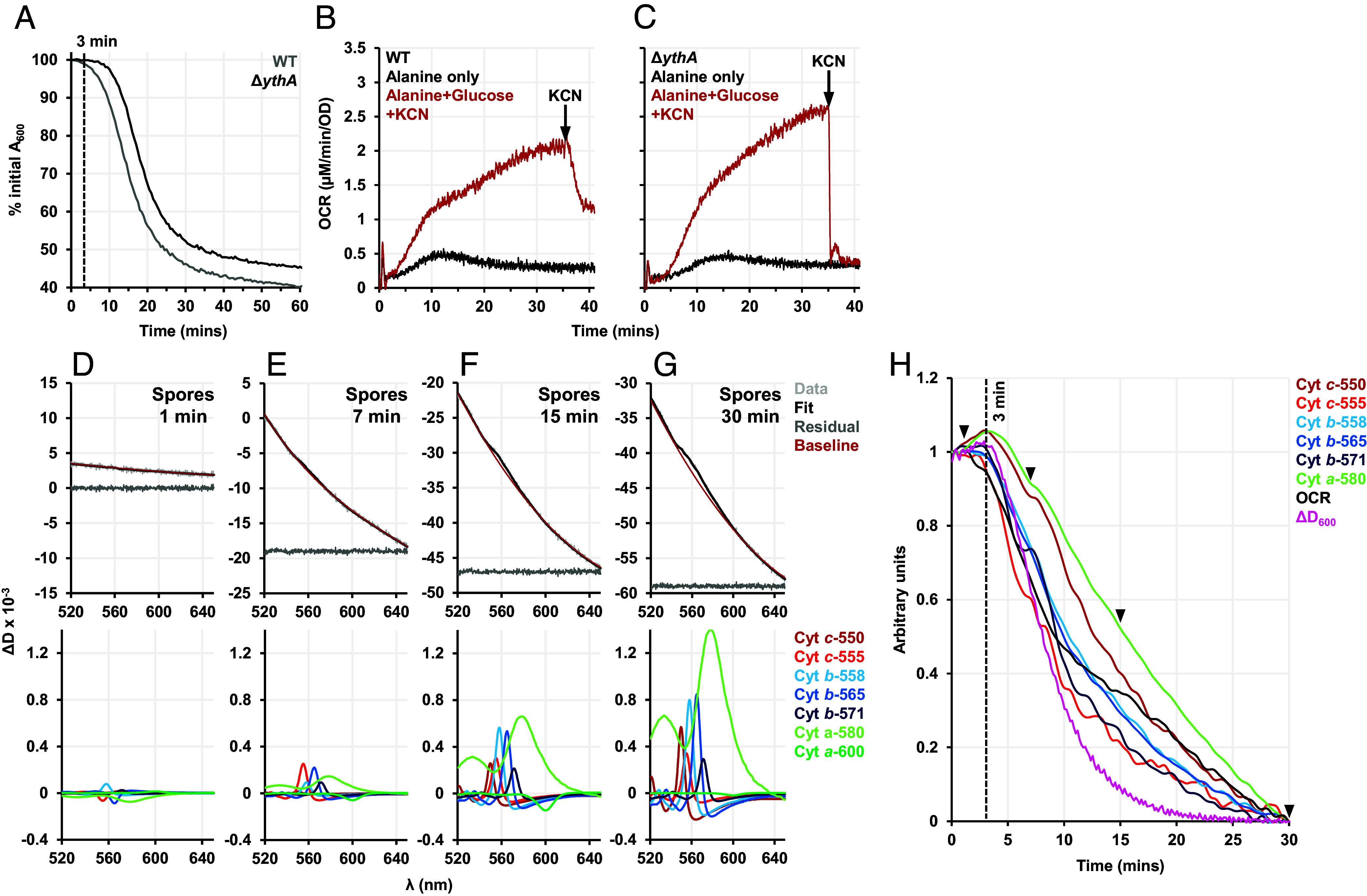
Translation of findings from *B. megaterium* in the spore-former *B. subtilis*. (*A*) Germination curves measured for the WT (gray) and Δ*ythA* (black) spores with 10 mM alanine. (*B* and *C*) OCRs measured for WT and Δ*ythA* spores germinated with 10 mM alanine alone (black) and 10 mM alanine+glucose (red). 1 mM KCN was added 35 min after alanine+glucose. (*D*–*G*) Remission spectra of spores germinated with 10 mM alanine+glucose. The *Top* panel shows the raw data, the fit imposed, the residuals (suitably offset from 0 for clarity), and the sum of the fitted baseline components. The *Bottom* panel shows the fitting of the haem components. (*H*) Savitzky–Golay smoothed and normalized kinetic traces of haem components, change in attenuance (ΔD_600_) and OCR for spores germinated with 10 mM alanine+glucose. Arrowheads indicate the time points 1, 7, 15, and 30 min for which the decomposition analysis is demonstrated in (*D*–*G*).

Next, we attempted to reproduce these findings in WT and Δ*ythA B. subtilis*. The Δ*ythA B. subtilis* spores were slower to initiate absorbance/attenuance loss than WT with 10 mM alanine (Fig. 5*A*). However, when germination was initiated with alanine only in the bioenergetic chamber, neither strain was able to achieve an appreciable OCR (Fig. 5 *B* and *C*). The addition of equimolar glucose along with alanine led to a considerably higher OCR and more pronounced spectral changes in both strains. As expected, O_2_ consumption was abolished by KCN in Δ*ythA B. subtilis* spores as flux is forced to the CN^-^-sensitive *aa*_3_-type oxidases (Fig. 5*C*).

In *B. subtilis*, the changes in attenuance were slower and approximately half that measured for *B. megaterium* spores at the same spore density (Fig. 5 *D*–*G*). The baseline fitting for *B. subtilis* spores was also markedly different to that for *B. megaterium* spores. A reason for this difference could be that these spores are distinct in their aspect ratios, with *B. subtilis* being more elongated (55). Scattering processes will differ depending on spore morphology as the wavelength of the light used and the spore sizes are of the same order (~1 μm) (56). Despite the differences, the unmixing model was sufficiently flexible to work well for *B. subtilis* spores, as judged by low residuals (Fig. 5 *D*–*G*). In *B. subtilis*, loss of ΔD_600_, haem reduction, and O_2_ consumption all start simultaneously. Intriguingly, compared to *B. megaterium*, OCR in *B. subtilis* started increasing earlier concomitantly with haem reduction, and the F-intermediate built much slower (Fig. 5*H*).

Outgrowth in Δ*ythA B. subtilis* spores progressed as in WT spores (*SI Appendix*, Fig. S2). Germinating *B. subtilis* spores were less reliant on Yth either because ΔΨ was lower, or the balance of the two *aa*_3_-type oxidases favored Qox, which is better “powered” to overcome the backpressure of ΔΨ than Cta (57). The *B. subtilis* Δ*ythA* spores outgrowing in nutrient broth could have also used metabolites that decreased their dependence on oxidative metabolism. Conversely, *B. megaterium* spores were more sensitive to perturbations of the higher yield oxidative pathway because they must conserve more energy to build a bigger vegetative cell.

## Discussion

**Fig. 6.**
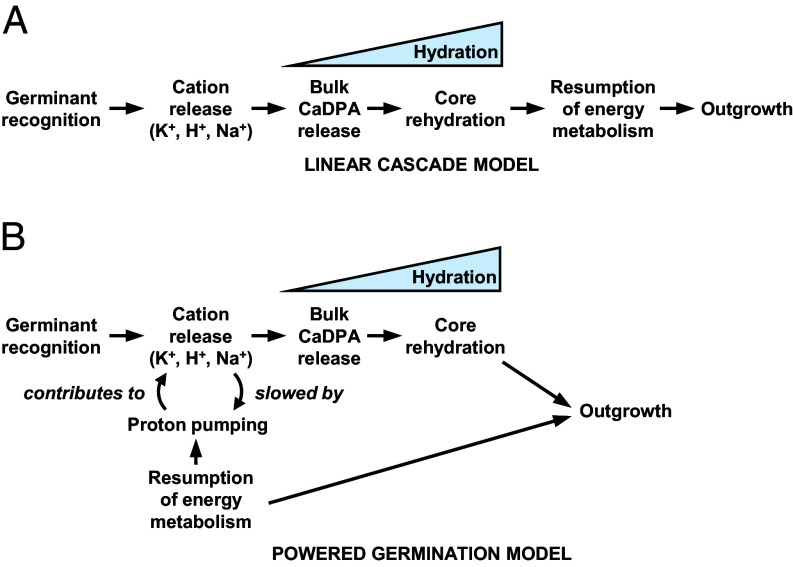
The linear cascade and powered germination models of *Bacillus* spore germination. (*A*) The linear cascade model where energy metabolism is resumed only after spore rehydration is completed. (*B*) The powered germination model based on the findings in this work. A nutrient germinant initiates Ger-mediated signaling (cation efflux) and is imported into the spore where its catabolism powers the resumption of electron transport and proton pumping.

Our results here lead us to propose the powered germination model (Fig. 6). In this model, germinants can act both as a signal and an energy source that is immediately exploited. For germinants to act as energy sources before or during rehydration, the gel-like core of a germinating spore must be able to support cytoplasmic metabolism. In *B. megaterium*, cations are released upon glucose recognition by GerU; glucose is also imported into the core where it is metabolized, passing electrons to the ETC. In *B. subtilis*, glucose acts as an energy source as in *B. megaterium*, and the GerA receptor recognizes alanine as the signaling molecule. In both cases, cation efflux and glucose-powered proton pumping cause a substantial ΔΨ to be rapidly established, which causes the accumulation of the F-intermediate of *aa*_3_-type oxidases. The substantial ΔΨ requires that these spores possess Yth; as Yth does not pump protons it will be much less sensitive to ΔΨ, allowing the oxidative metabolism of glucose even when *aa*_3_-type oxidases are hindered by ΔΨ. The receptor-mediated signaling cascade and bioenergetic processes are semi-independent as demonstrated by the deletion of Yth slowing and anaerobiosis accelerating loss of absorbance/attenuance. Recent orthogonal studies support our model. In *B. subtilis*, luciferase-induced luminescence, which correlates with ambient cellular redox activity, was synchronized with phase transition (58). In *B. atrophaeus*, O_2_ slowed germination but was essential for subsequent colony formation (59). Before now, germinating spores have been considered de-energized, with molecular processes of germination (e.g., cation/CaDPA release, water ingress) running spontaneously downhill. From a purely bioenergetic point of view, our findings allow powered processes (e.g., active transport, macromolecular synthesis) to be invoked during germination but with the caveat that while energy availability may not be limiting, other factors can be.

Recently, there has been renewed interest in cation efflux during spore germination (16, 29). While seen as signaling events, such charge movements also conserve energy. Proton pumping by the *aa*_3_-type oxidases will contribute to both the PMF for ATP synthesis and the charge imbalance created by Ger receptors (Fig. 6*B*). In *B. megaterium*, there is little O_2_ consumption until cytochrome reduction is well underway, so proton pumping will contribute less to the Ger-mediated cation efflux, but in *B. subtilis*, O_2_ consumption starts relatively early alongside cytochrome reduction, so proton pumping can contribute directly to the cation gradient and in turn be influenced by it.

ATP accumulation has been measured in extracts from germinating spores (25–27). These studies found that ATP accumulated once detectable germination had started, inferring that bioenergetic processes must also restart around then (60). However, the early generation of ΔΨ through cation efflux along with the abundance of ATP synthase in the dormant spore membrane ought to create conditions for ATP synthesis from an early time point. Whether ATP can power reactions in the “forward” direction will depend on the ΔG of the ATP-hydrolysis reaction (the phosphorylation potential) and the ΔG of the reaction being powered, both of which will probably be considerably different in spores compared to vegetative cells. Additionally, the concentration of ATP/ADP/P_i_ alone can give a false impression of an inactive bioenergetic system if synthesis of ATP is high but consumption is even higher, particularly if the system starts from a de-energized state. While ATP/ADP/P_i_ levels have been quantified for dormant spores (23, 25), measurement from germinating spores relies on ATP/ADP/P_i_ extraction without their concentrations being affected by enzymatic side-reactions, which is technically challenging to control for. Noninvasive measurements on intact germinating spores using techniques such as ^31^P-NMR may be powerful here (61).

The glycolytic intermediate 3-phosphoglyceric acid (3-PGA) is thought to be the major endogenous energy reserve that spores use during germination as substrate-level phosphorylation of 3-PGA-derived PEP generates ATP. Spores germinated in nutrient-free media with KBr (25) or CaDPA (27) are reliant on this pathway. Indeed, in the inverse process by which dormancy is established, recent modeling suggests that the forespore generates ATP by substrate-level phosphorylation of glycolytic intermediates provided by the mother cell (62). However, spores germinated with physiological germinants will have a substantial exogenous energy supply to be exploited for higher ATP yields by oxidative phosphorylation.

We speculate that the potency of a germinant can be partially linked to the capacity a germinating spore has to directly use it, e.g., glucose is rapidly catabolized by the gluconate pathway in *B. megaterium* to generate reducing power. In *B. subtilis*, alanine alone leads to rapid germination but relatively low O_2_ consumption and meager cytochrome reduction, yet Δ*yth* slows alanine-initiated germination because alanine transamination/oxidation and gluconeogenesis would also subsequently lead to ETC reduction (63). Alanine is ubiquitously recognized as a germinant by Bacillus and Clostridial spores and even some fungi, suggesting it is a common ancestral germinant (2, 64). Alanine is a reliable environmental signal for many of these spores but may not be as readily metabolizable by existing pathways, which in *B. subtilis* also favor the cogerminant glucose as the source of reducing power (65).

While the relationship between bioenergetics and spore germination was actively explored early in the field, technical limitations of quench-and-measure approaches meant that bioenergetics were overlooked and discounted. Here, we have noninvasively measured bioenergetic events in spores responding to physiological germinants in real time. We focused on two model species but anticipate that similar principles will apply to many other spore-formers.

## Materials and methods

### Bacterial Strains and Culture Conditions.

All strains used in this work are listed in *SI Appendix*, Table S3 along with their genotypic/phenotypic description. The Δ*ythA* mutants were isogenic with the wild-type *B. megaterium* QM B1551 and *B. subtilis* 168 strains. All *Bacillus* strains were routinely cultured in LB medium supplemented with appropriate antibiotics when warranted (erythromycin at 0.5 or 1 μg.mL^−1^; lincomycin at 12.5 or 25 μg.mL^−1^; kanamycin at 5 or 10 μg.mL^−1^; tetracycline at 12.5 μg.mL^−1^), and incubated at 30 °C for *B. megaterium* or 37 °C for *B. subtilis*. Plasmids to generate the Δ*ythA B. megaterium* mutant were propagated and isolated from NEB Turbo *Escherichia coli* (New England Biolabs, UK) cultured at 37 °C in LB medium supplemented with carbenicillin (100 μg.mL^−1^). The *B. subtilis* WT and Δ*ythA* spores were obtained from the Bacillus Genetic Stock Center library (66).

### Mutant Strain Construction.

An insertion-deletion in the *ythA* gene (BMQ_4878) of *B. megaterium* QM B1551 was produced by allelic exchange. The plasmid used was assembled from the following, all containing 20 bp overlaps: upstream (positions 4,684,943 to 4,685,552; KEGG *B. megaterium* QM B1551 genome) and downstream (positions 4,685,727 to 4,686,337; KEGG *B. megaterium* QM B1551 genome) regions of BMQ_4878, kanamycin (Km) resistance cassette from pDG792 (67), and backbone fragments from pUCTV2 to obtain a temperature sensitive origin of replication and a tetracycline (Tet) resistance cassette (68). PCR products were electrophoresed on agarose gels, extracted under blue light illumination, and purified (QIAquick Gel Extraction Kit, Qiagen, UK). Resultant fragments were combined using an in-house Klenow Assembly Method at 37 °C for 45 min, with 2 μL of the subsequent product used to transform Turbo *E. coli* (NEB, UK) to carbenicillin resistance. Transformant *E. coli* was harvested and the purified plasmid (QIAprep Spin Miniprep Kit, Qiagen, UK) was verified by sequencing.

Plasmid pUCTV2-ΔBMQ_4878::Km was introduced into *B. megaterium* QM B1551 by polyethylene glycol (PEG)-mediated protoplast transformation, and recovered on RHAF agar containing 5 μg.mL^−1^ Km at 30 °C overnight (69). Transformant colonies were dotted in a grid-like fashion on LB agar containing 5 μg.mL^−1^ Km at the nonpermissive temperature of 42 °C overnight, to enable integration of the plasmid at the cloned locus via homologous recombination. Subculture of the resultant single crossover *B. megaterium* was continued at 42 °C on LB agar lacking antibiotics until the desired double-crossover colony was isolated. Successful double-crossover resulted from the excision of pUCTV2 plasmid (Tet^s^) and disruption of BMQ_4878 because of ΔBMQ_4878::Km integration (Km^r^). Colony PCR confirmed the double-crossover mutant but also revealed loss of the native pBM700 plasmid carrying the GerU germinant receptor. To restore the GerU-mediated germination response to glucose, the episomal pHT315-GerU* plasmid (70) was introduced by PEG-mediated protoplast transformation into *B. megaterium* ΔBMQ_4878::Km on RHAF (1 μg.mL^−1^ erythromycin + 25 μg.mL^-1^ lincomycin) at 30 °C overnight. Colony PCR was performed to confirm the genotypes of WT and ΔBMQ_4878 strains (Fig. 4*A*). The primers used are listed in *SI Appendix*, Table S4. The *gerUA* product with primer pair p106+p107 at 502 bp was obtained for both WT and ΔBMQ_4878 strains as expected. Due to the insertion of the Km cassette, a bigger product was obtained at 3,219 bp with primer pair p114+p115 for the ΔBMQ_4878 strain, compared to the 1,905 bp product obtained for WT.

### Spore Cultivation and Purification.

*B. megaterium* spores were prepared by nutrient exhaustion in 500 mL of supplemented nutrient broth (SNB) medium (71) in baffled flasks shaken at 225 rpm, 30 °C, for 72 h. For the Δ*ythA* mutant, the SNB medium was supplemented with 0.5 μg.mL^−1^ erythromycin to maintain the pHT315-GerU* plasmid. The spores were washed five to six times (4 K RCF, 4 °C, 10 min) with ice-cold sterile water until 99% purity was achieved, as confirmed by phase contrast microscopy. The pellet was resuspended in 5 mL sterile water and stored on ice in the cold room at 4 °C.

Washed spores were subjected to further purification using a Histodenz density gradient (72). 250 μL of the washed spore suspension was centrifuged and the pellet resuspended in 200 μL 20% Histodenz (Sigma-Aldrich), which was layered on top of 1.2 mL 60% Histodenz in a 2 mL microcentrifuge tube. This was centrifuged at 16 K RCF for 15 min at 4 °C, after which the supernatant containing vegetative debris was carefully removed. The purified spore pellet was resuspended, transferred to a fresh tube, and washed three to four times with ultrapure water at 4 K RCF, 4 °C to remove residual Histodenz after which the purified spores were resuspended in 250 μL ultrapure water. The OD (optical density at 600 nm) of such Histodenz-purified spore suspensions varied between 200 and 400. Purified spores were always stored on ice and all experiments were performed within 10 d of spore harvest.

*B. subtilis* spores were also prepared by nutrient exhaustion in 2× Schaeffer’s-glucose (2×SG) agar medium (73), incubated at 37 °C for 72 h. For the Δ*ythA* mutant, the 2×SG medium was supplemented with 5 μg.mL^−1^ kanamycin. The spores were scraped off the plates, washed five to six times (18 K RCF, 4 °C, 5 min), and resuspended in 3 mL sterile water for storage on ice in the cold room at 4 °C. They were later purified using a Histodenz density gradient in the same way as described for *B. megaterium* spores, except that 50% Histodenz was used instead of 60% Histodenz, and the Histodenz-purified spores were stored at an OD of 150.

### Spore and cell membrane isolation and protein quantification.

Membranes were isolated by disrupting spores (from 2 L culture) that were washed thoroughly but not purified further using gradient centrifugation. The washed spores were first treated with 0.1 M NaCl, 0.1 M NaOH, 0.1 M DTT, and 0.5% SDS at 37 °C for 1 h to chemically remove the protein coat and the outer membrane. The decoated spores were washed five to six times with ice-cold dH_2_O (4 K RCF, 4 °C, 10 min), and resuspended in fresh buffer (50 mM Tris-SO_4_ pH 7.5, 50 mM NaCl), supplemented with 1 mg.mL^−1^ lysozyme (Roche diagnostics), 0.01 mg.mL^−1^ DNase I (Sigma-Aldrich), 5 mM MgCl_2_, and 1 cOmplete™, Mini protease inhibitor cocktail tablet (Roche diagnostics). This enzymatic treatment was carried out at 4 °C for 45 min with gentle agitation primarily to degrade the peptidoglycan cell wall and cortex, before mechanical disruption of the spores by four to five passages through a cell disrupter (Constant Systems Ltd.) at 30 KPSI to fragment the degraded cortex/membranes and release the core contents. Next, the spore lysate was clarified by centrifugation at 50 K RCF, 4 °C for 20 min to remove unbroken spores and integument fragments, and the supernatant was ultracentrifuged at 150 K RCF, 4 °C overnight (Beckman). The spore membranes were obtained as a red-orange pellet while the supernatant had a yellow coloration. The isolated membranes were washed twice using buffer containing 50 mM Tris-SO_4_ pH 7.5, 50 mM NaCl (150 K RCF, 4 °C, 1 h), homogenized using a Dounce homogenizer (Kimble), aliquoted, and stored at −70 °C. The supernatant was syringe-filtered through a 0.22 μm membrane (Sartorius) and concentrated down from ~50 mL to 5 mL using a 30 kDa MWCO centrifugal concentrator (Sartorius), then aliquoted and stored at −70 °C.

Membranes from *B. megaterium* late-exponential phase cells (16 h of growth in LB broth medium, 30 °C, 225 rpm shaking incubation) were isolated using a similar method: cell lysis in the presence of 1 mg.mL^−1^ lysozyme and 0.01 mg.mL^−1^ DNase I, 5 mM MgCl_2_, and 1 protease inhibitor tablet followed by passage through a cell disruptor twice at 30 KPSI. The lysate was centrifuged (10 min, 10 K RCF, 4 °C) four times to pellet any debris and the membranes isolated from the supernatant by ultracentrifugation (150 K RCF, 4 °C, 1 h). The isolated membranes were washed twice (1 h, 150 K RCF, 4 °C), aliquoted, and stored at −70 °C.

The protein concentration was measured using the Bicinchoninic acid (BCA) protein assay kit (Sigma-Aldrich) following the recommended protocol. BSA (2 mg.mL^−1^) was used to make standards and a dilution series was prepared for the membrane preparations (1/10, 1/20, 1/40, 1/80, 1/160, 1/320, 1/640, and 1/1,280). 25 μL standards/sample dilutions were pipetted in triplicate into a 96-well plate followed by 200 μL BCA reagent added to each well. The plate was then incubated at 37 °C for 30 min. Absorbance was measured at 562 nm in the SpectraMax ABS Plus microplate reader (Molecular Devices) and the data analyzed using a predefined protocol in Softmax Pro 7.1.

### Remission Haem Spectroscopy and Oxygen Consumption Measurements.

Remission haem spectroscopy experiments on cells, spores, and isolated membranes were performed with the prototype of the Iberius Cell Spectroscopy System (CellSpex Ltd.) which consisted of a bioenergetic chamber, a single-channel spectroscopy system and an oxygenation system. The bioenergetic chamber had a sample volume of 5 mL. 3.5 mL of *B. megaterium* cells grown in 5 mL LB medium to an O.D. of ~5.4 (exponential phase, incubated for 12 h) were diluted with 1.5 mL fresh LB medium to achieve an O.D. of 4. Isolated membranes from cells and spores were diluted in the buffer containing 10 mM Tris-SO_4_ pH 7.3 and 250 mM sucrose such that the final protein concentration was 0.25 mg.mL^−1^. Histodenz-purified and heat-shocked spores were resuspended in 5 mL 50 mM potassium phosphate pH 7.5 buffer to obtain an O.D. of 6. A white LED light source, Luxeon CZ 4000 K-90 (Lumileds) used at a current of 200 to 350 mA, illuminated the samples in a quartz crucible. The backscattered light was collected in remission geometry, passed through a spectrograph (iHR320, Horiba) equipped with a 300 g/mm grating blazed at 500 nm, and complete spectra between 480 and 760 nm were collected on a CCD camera (Andor Technology). The slits were set to 100 μm to give a spectral resolution of 1 nm. The O_2_ optode relies on the phosphorescence half-life of a platinum-porphyrin compound to measure O_2_ concentration (74). In all experiments with intact spores except one (Fig. 3*F*), a blend of N_2_/O_2_ was delivered through silicone tubing (Braintree Scientific. Inc.) submerged in the samples to maintain a constant O_2_ concentration throughout (40). OCR was calculated from the difference between the O_2_ delivery to the sample and the rate at which the O_2_ concentration in the sample changed during the experiment (32, 34).

To prepare the device for an experiment, a wavelength calibration for the CCD was performed using mercury emission lines (546.074, 576.960, and 579.070 nm), and the optode was calibrated by measuring O_2_ dissolved in the experimental buffer exposed to air at the desired temperature vs. the zero-point achieved by the addition of the strong reducing agent sodium dithionite (Na_2_S_2_O_4_). Then, with the sample buffer in the crucible, tubing in place, and the chamber sealed, the intensity of the LED light source was calibrated (serving as a blank measurement). Following the addition of the cell/isolated membranes/spore samples, they were allowed to equilibrate for 10 to 15 min in the re-sealed chamber. The experiments were initiated by the addition of a germinant/NADH/KCN via a small injection port present in the plunger seal at t = 0 min and one spectrum was recorded every 20 ms in two phases (each phase is 10 ms long). The device was controlled using the accompanying Palencia software (CellSpex Ltd.).

The phosphorescent light at 650 nm from the O_2_ optode can interfere with attenuance measurements in this wavelength range. The interference was removed using two-phase time multiplexing: The samples were illuminated with a white LED in the first phase but not the second. The spectrum from the second phase was then subtracted from that of the first phase to remove the signal from the O_2_ optode which was present in both phases. Each phase was 10 ms and spectra from 25 pairs of phases were averaged to give a temporal resolution of 500 ms.

The spectra were analyzed and manipulated (generation of averaged difference spectra, scaling, Savitzky–Golay smoothing, offsetting) in the Gerona analysis software package (CellSpex Ltd.). The difference spectrum at a given time point T_p_ was generated by averaging the 120 spectra recorded over a 1-min interval preceding the T_p_. From this, the spectrum before glucose/CN^-^ addition was subtracted, and the resulting spectra were fitted to a linear regression to generate the difference spectra shown. Difference spectra for isolated membranes were generated by subtracting the averaged air-oxidized spectrum from the averaged Na_2_S_2_O_4_-reduced spectrum. A linear regression was further subtracted from these difference spectra as baseline correction.

Where the Savitzky–Golay smoothing function (75) was used, the half width was 0.5 nm and the order was 1. Decomposition of the haem attenuance spectra was performed using the following equation implemented in Gerona as the “FIT:NIR” model:

$$C=\frac{1}{\rho }(S^{T}S)S^{T}A,$$

where C is the unknown column matrix containing the concentration of each component, ρ is the differential pathlength, A is the column matrix containing the observed attenuance at each wavelength, and S is the known matrix containing the specific absorbance of each haem center at each wavelength (40, 47, 76). Model spectra for the fitting template were taken from various sources, wavelength-shifted to get the desired peak absorbance, and normalized to 1. The spectra used to generate the cyt *c*-550, *c*-555, *b*-558, *b*-565, and *b*-571 model spectra were originally measured using beef heart cyt *bc*_1_ complex. The Cyt *a*-580 spectrum was obtained from ref. 77, and is the spectrum of the fully reduced *B. subtilis aa*_3_-600 with O_2_ at a high pH after 120 min. Cyt *a*-600 is the haem *a* difference spectrum taken from ref. 78. The baseline components used were α = 1/x^2^, β = (x − 585), γ = (x − 585)^2^, and δ = 1, where 585 nm is the midpoint of the fitting range (520 to 650 nm).

### Spore Germination and Outgrowth Assays.

Spore germination and outgrowth were assessed by monitoring the change in their initial absorbance at 600 nm. Histodenz-purified *B. megaterium* spores were heat shocked either at 60 °C for 10 min in a water bath or at 70 °C for 20 min in a heating block. Histodenz-purified *B. subtilis* spores were heat shocked at 70 °C for 30 min in a heating block. The heat-shocked spores were either cooled on ice or washed once (4 K RCF and 18 K RCF for *B. megaterium* and *B. subtilis* spores, respectively, 4 °C, 10 min) and resuspended in fresh 50 mM potassium phosphate pH 7.5 buffer. In a 96-well plate with the germinants/nutrient broth/KCN and combinations thereof already present in triplicates, the spore suspension was added to start the assay such that the initial absorbance was 0.6 to 0.8 in a 200 μL volume. Absorbance measurements at 600 nm were then started immediately in a SpectraMax ABS Plus microplate reader (Molecular Devices) or a Tecan Infinite-200 series monochromatic plate reader fitted with a 600-nm photometric filter. They were set at 30 °C and 37 °C, with a read interval of 10 s and 30 s for *B. megaterium* and *B. subtilis* spores, respectively. Where specified, assays were performed either with or without orbital shaking for 10 s between absorbance readings. Germination/outgrowth assays under anaerobiosis were carried out in an anaerobic/dry glove box system (Belle Technology UK Ltd.) maintained at 2.5 ppm (0.00025%) O_2_ with a SpectraMax ABS Plus microplate reader inside. The data were imported from the microplate reader software SoftMax Pro 7.1 and i-control 2.0 into Microsoft Excel and the absorbance values across the technical replicates was averaged. Absorbance measured at t = 0 min (t_0_) was taken as the initial absorbance, and the percentage loss of initial absorbance was calculated for all subsequent time points. The % initial A_600_ values were plotted against time to give germination and outgrowth curves. The rate of germination was given by the first derivative of % loss (t_p_) values. This was calculated using a window of 11 data points and the function SLOPE which returned the slope of the line at the center of the window as the window moved to the next data point. The first derivative (% loss of initial A_600_/min) was then plotted against time. Experiments were conducted with at least two biological replicates (independently prepared spore batches).

### Glucose Consumption Assays.

Glucose consumption of germinating *B. megaterium* spores was inferred from the enzymatic quantification of glucose remaining in the germination medium. Pellets of heat-shocked spores were resuspended in 285 μL 50 mM potassium phosphate pH 7.5 buffer and transferred to a 2 mL microcentrifuge tube. All tubes were moved to a heating block set at 30 °C, 300 rpm shaking, with their lids open. Germination was initiated with the addition of 15 μL 20 mM glucose (final concentration of 1 mM in 300 μL volume) and the spores (O.D. of 17) were incubated for the stipulated period (t = 0, 2, 4, 6, 8, 10, 12, 14, 16 min) after which they were quickly relocated to a heating block set at 100 °C for 10 min to stop germination, then put in ice until all samples were ready. The tubes were then centrifuged at 16 K RCF, 4 °C for 15 min. The supernatants were transferred to fresh tubes and centrifuged again to remove all the spore debris. The D-Mannose/D-Fructose/D-Glucose assay kit (Megazyme Ltd.) was used for the quantification of glucose in these supernatants along with the 1 mM glucose control (without any spores) with minor modifications to the recommended kit protocol. Stoichiometric amounts of NADPH were formed in a 126 μL reaction volume containing 50 μL of each sample. This was performed in triplicates for each time point and the control. The triplicate values from the glucose assay were averaged for each time point and the control, and the amount of glucose consumed (μM) by t minutes was calculated and normalized for O.D. of 17. Glucose consumed (μM/O.D.) values were plotted against time (min), and a linear regression was fitted in the 6- to 16-min region of the curve to get an average rate of glucose consumption. Experiments were conducted with at least two biological replicates (independently prepared spore batches).

### Hydrogen Peroxide Production Assay.

Hydrogen peroxide released by spores germinated with 1 mM/4 mM glucose was measured using an Amplex Red assay kit (Invitrogen, ThermoFisher Scientific). 200 μL reactions were set up in duplicate with heat-activated spores (1 O.D.), 0.5 U.mL^−1^ horseradish peroxidase (HRP) and 50 μM Amplex Red reagent in a 96-well flat-bottom black microplate (Nunc, ThermoFisher Scientific). The negative control reaction, also used for background subtraction during data analysis, contained spores, HRP, and Amplex Red but no glucose. The reagent injectors present in the CLARIOstar microplate reader (BMG Labtech) were used to dispense 10 μL of the 20 mM/80 mM glucose stocks to initiate germination, which was immediately followed by double orbital shaking at 300 rpm for 30 s, then fluorescence measurements every 15 s for 30 min at 30 °C. The excitation/emission wavelengths used were (545-20)/(600-40) which are preset for the reaction product resorufin in the control software, number of flashes/well = 20, gain = 744 and focal height = 8.2 mm. H_2_O_2_ standards were prepared by serial dilution and reactions were set up in triplicate containing the standard, 50 μM Amplex Red and 0.2 U.mL^−1^ HRP. A single-point measurement using the same optic settings was recorded in the microplate reader, and the blank-subtracted fluorescence values were used to plot a standard curve. The blank-subtracted fluorescence values were used to calculate the H_2_O_2_ concentration (nM). The rate of H_2_O_2_ release was given by the first derivative calculated using a window of 5 data points. This first derivative (nM/min) was then plotted against time. Experiments were conducted with at least two biological replicates (independently prepared spore batches).

### BN-PAGE and LC/MS Analysis.

BN-PAGE analyses were performed using the well-established NativePAGE Novex Bis-Tris Gel System (Invitrogen, ThermoFisher Scientific). The spore membranes at a protein concentration of 4 mg.mL^−1^ were solubilized with 1% n-dodecyl β-D-maltoside (DDM, GLYCON Biochemicals GmbH) with gentle agitation for 1 h at 4 °C. The extracted proteins were clarified twice at 16 K RCF, 4 °C for 15 min. The sample was resolved using a precast 3 to 12% Bis-Tris gradient gel, along with the NativeMark Unstained Protein Standard in a 4 °C cold room at 150 V for the first 60 min and at 250 V for the last 30 to 45 min. The gel was later rinsed with dH_2_O, and fixed for 10 min in a mixture of 50% ethanol and 10% acetic acid on a gel rocker. After thorough destaining in dH_2_O, the gel was submerged in the staining solution containing Coomassie brilliant blue G-250 dye and HCl, heated, and left to stain overnight on the gel rocker—this was needed to stain the low abundance membrane proteins for maximum contrast. The following morning, the gel was destained again. Once sufficiently destained, the gel was imaged. Bands were cut out for analysis by the Metabolomics & Proteomics Lab, Technology Facility, Department of Biology, University of York using Liquid Chromatography tandem Mass Spectrometry (LC/MS). A Waters mClass UPLC connected to an Orbitrap Fusion Tribrid mass spectrometer was used for data acquisition, and Progenesis QI was used for chromatographic alignment and peak picking. The protein hits in each BN-PAGE band were then sorted (largest to smallest) based on their peak areas.

### Phase Contrast Microscopy.

Microscopic examination was performed in tandem with the outgrowth assay. 2 μL of the spore sample from the appropriate well before and during the outgrowth assay were air dried on a glass slide. A coverslip was placed on the nearly dried drop and pressed down to expel air pockets. An Olympus BX53 microscope fitted with a QImaging Retiga 2000R CCD camera microscope, controlled with the software Q-Capture Pro 7 was used to obtain phase contrast images using a 100× objective lens.

## Supplementary Material

Appendix 01 (PDF)

Dataset S01 (XLSX)

## Data Availability

All study data are included in the article and/or supporting information.
