# Supplementary material for: Early activation of bioenergetic metabolism powers bacterial spore germination
Source: Proc Natl Acad Sci U S A. 2025 Dec 24;122(52):e2510996122. doi: 10.1073/pnas.2510996122 (PMC12772218; doi:10.1073/pnas.2510996122)
Supplement: Supplementary file 1 — Appendix 01 (PDF) [file pnas.2510996122.sapp.pdf]

PNAS

Supporting Information for

Early activation of bioenergetic metabolism powers bacterial spore germination

Pooja Gupta[1,2ϯ], Rebecca Caldbeck[3], Rowan C Walters[1,2], Elodie C Wells[1,2✧], Bethany L Hardman[1,2^], Graham Christie[3], Roger J Springett[1,2,4], James N Blaza[1,2*]

[1] York Structural Biology Laboratory, Department of Chemistry, University of York, York, UK
[2] York Biomedical Research Institute, University of York, York, UK
[3] Department of Chemical Engineering and Biotechnology, University of Cambridge, Cambridge, UK
[4] CellSpex Ltd, Northampton, UK

ϯ Present address: University of Oxford, Oxford, UK ✧ Present address: University of Southampton, Southampton, UK ^ Present address: University of Durham, Durham, UK

Email: jamie.blaza@york.ac.uk

This PDF file includes:

Supporting text
Figures S1 to S3
Tables S1 to S4
Legend for Dataset S1
SI References

Other supporting materials for this manuscript include the following:

Dataset S1 (separate file). All proteins identified in the LC/MS analysis of BN-PAGE bands 1-6 (Fig 1F), and the GDH-enriched 'GDH-6' fraction (Fig S1).

Supporting Information Text

Supplementary methods

NADH oxidation assays

The NADH oxidation rates for the membrane preparations were measured spectrophotometrically. The reaction buffer used was 10 mM Tris-SO_4 pH 7.3 and 250 mM sucrose in a reaction volume of 200 µL in a flat-bottomed 96-well plate containing 200 µM NADH and 0.05 or 0.1 mg.mL^{-1} protein. The volume of the buffer was adjusted accordingly when 1 mM KCN was added. Data were recorded at 340-380 nm every 7 secs for 20 mins in the SpectraMax ABS Plus microplate reader maintained at 30 °C. Rates for the linear region of the oxidation curves were calculated in the software Softmax Pro 7.1. $\varepsilon_{340\text{-}380nm}$ = 4810 M^{-1}cm^{-1} used for the calculations (1).

Chromatographic enrichment of GDH activity

The supernatant obtained after overnight ultracentrifugation of the spore lysate contained soluble proteins present in the core spore. This was syringe-filtered through a 0.22 µm membrane and concentrated down from ~50 mL to 5 mL using a 30 kDa MWCO centricon and stored at -70 °C. 500 µL of the whole spore soluble fraction was first subjected to SEC (Superdex 200 Increase 10/300 GL, 8 °C), then AEX (HiTrap Q FF, 21 °C) and lastly to SEC again (Superdex 200 Increase 3.2/300 GL, 4 °C). Following each of these chromatographic steps, the resulting fractions were screened for GDH activity using a spectrophotometric assay at 30 °C in a 96-well plate format. 50/5 µL of each SEC fraction was added in a 200 µL reaction also containing 100 µM NAD^+ and 1 mM glucose. These reactions were performed in a 50 mM Tris-SO_4 pH 7.5, 50 mM NaCl buffer and absorbance at 340-380 nm was measured every 15 secs or 6-7 secs for 30 mins. The protein concentration of SEC/AEX fractions was also measured with single-point readings using a Bicinchoninic acid (BCA) protein assay kit (Sigma-Aldrich) following the recommended protocol. Dilution factors of 2.5 (after SEC I), 25 (after AEX) and 12.5 (after SEC II) were used – 25 µL of the diluted samples was used for assay as described, and the value obtained was multiplied by the dilution factor to get the protein concentrations of the SEC/AEX fractions. These rates were then plotted against the corresponding fraction number to visualise regions of enriched GDH activity. The fraction with the highest GDH activity was analysed by LC/MS as described in the main text.

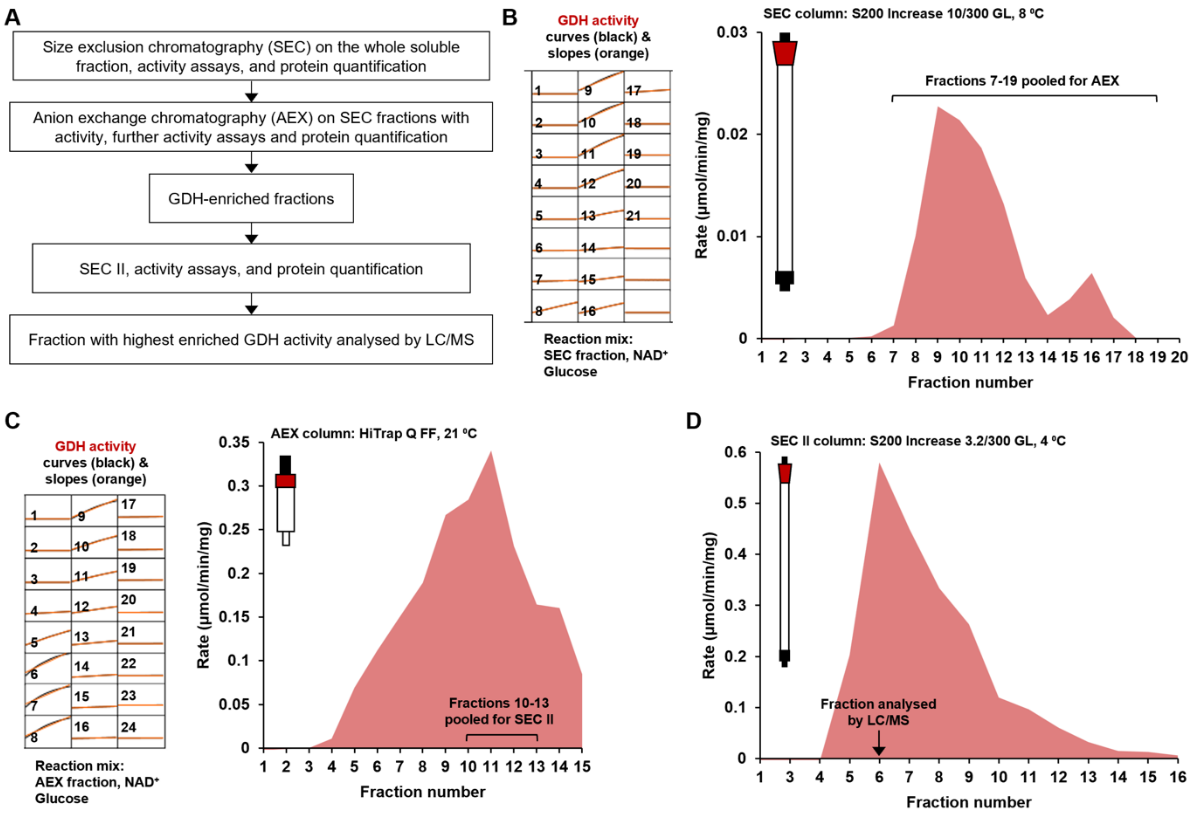

Fig. S1. Enrichment of GDH activity from the whole spore soluble fraction. (A) A flowchart summarising the experimental strategy to enrich GDH activity. (B) Left panel shows curves (black) from the spectrophotometric GDH activity assay carried out for each SEC elution fraction, the slopes (orange) calculated by the plate reader software, and the components of the reaction. GDH activity would reduce NAD^+ to NADH, increasing the $A_{340-380}$. The graph on the right shows the rate of GDH activity measured for each fraction numbered 1-21. Based on this, SEC elution fractions 7-19 were pooled and subjected to AEX. (C) Same as (B) but with AEX elution fractions. 'GDH-enriched' AEX elution fractions 10-13 were pooled and subjected to another SEC run. (D) After the second SEC, specific GDH activity was enriched further. Fraction GDH-6 was saved for LC/MS analysis.

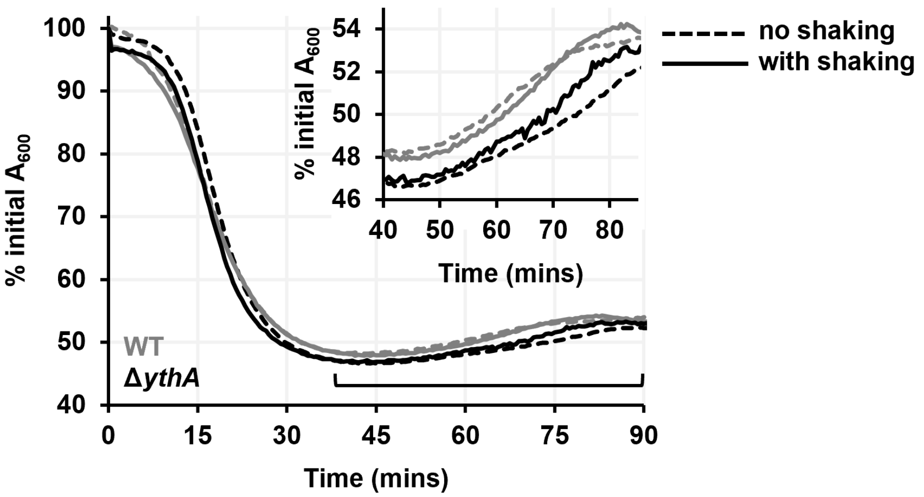

Fig. S2. Outgrowth curves for WT (grey) and Δ*ythA* (black) *B. subtilis* spores germinated with 10 mM alanine and nutrient broth, when the 96-well plate was shaken (solid line) or not (dashed line) during the experiment. Inset shows only the indicated outgrowth phase (40-90 mins) for clarity.

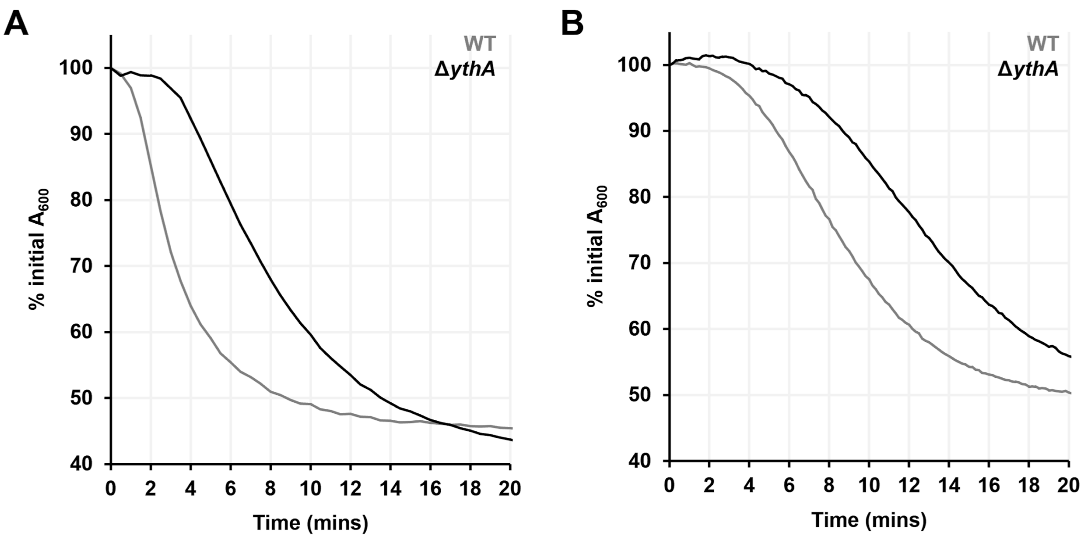

Fig. S3. Germination curves for WT (grey) and Δ*ythA* (black) spores of (A) *B. megaterium* and (B) *B. subtilis* both cultivated without antibiotics erythromycin/kanamycin. *B. megaterium* and *B. subtilis* spores were germinated with 10 mM glucose and 10 mM alanine, respectively.

Membranes	NADH oxidation rate (µmol/min/mg)	NADH oxidation rate in presence of 1 mM KCN (µmol/min/mg)	Fraction NADH oxidation lost in the presence of 1 mM KCN (%)
Vegetative Cells	0.15 ± 0.01	0.074 ± 0.002	51
Spore preparation 1	0.23 ± 0.01	0.20 ± 0.01	13
Spore preparation 2	0.22 ± 0.01	0.17 ± 0.01	23
Spore preparation 3	0.32 ± 0.01	0.25 ± 0.01	22

Table S1. NADH oxidation rates measured for isolated cell and spore membranes, and the effect of KCN which is an inhibitor of cytochrome aa_3-type oxidases.

Name of protein from UniProt	BMQ code	Unique peptides	KEGG/GenBank annotation
Glucose-6 phosphate isomerase	BMQ_4937	23	
Glucose 1-dehydrogenase (NAD(P)($^{+}$))	BMQ_0838	8	Glucose 1-dehydrogenase (gdh)
Glucose-6-phosphate 1-dehydrogenase	BMQ_5210	20	-
Glucose 1-dehydrogenase (NAD(P)($^{+}$))	BMQ_2939	5	short-chain dehydrogenase/reductase
PTS system, glucose-specific IIBC component	BMQ_1302	24	-
Glucose 1-dehydrogenase (NAD(P)($^{+}$))	BMQ_2333	2	-
Glucose-6-phosphate 1-dehydrogenase	BMQ_1958	7	-
Sugar phosphotransferase system, glucose subfamily, IIA component	BMQ_4019	3	-
Glucose 1-dehydrogenase (NAD(P)($^{+}$))	BMQ_2208	3	3-hydroxybutyrate dehydrogenase
UTP--glucose-1-phosphate uridylyltransferase	BMQ_5130	7	-
Glucose 1-dehydrogenase (NAD(P)($^{+}$))	BMQ_1269	4	oxidoreductase, short chain dehydrogenase/reductase family protein

Table S2. Most abundant proteins containing the word “glucose” in the GDH-enriched ‘GDH-6’ fraction identified by LC/MS.

Strain	Genotype/phenotype
QM B1551	Wild-type *B. megaterium*
Δ*ythA B. megaterium*	QM B1551 Δ*ythA*::Km pHT315-GerU* Δ*ythA*, MLS^{r*}
168 trpC2	Wild-type *B. subtilis*
Δ*ythA B. subtilis*	168 trpC2 Δ*ythA*::$Km^{r\#}$

*macrolide-lincosamide streptogramin-B resistance
#kanamycin resistance

Table S3. List of *Bacillus* strains used in this study.

Gene	Forward primer	Reverse primer
gerUA (BMQ_pBM70070)	p106: GCTACTGATCAATCCGCAGAACATATACAAGATTCG	p107: TAATGATGCGACGTACCATGGCTGTATTGGTTCC
ythA (BMQ_4878)	p114: GCGGATATGTGTTGCCATCTTCCCATTGAACGGTC	p115: GATACTTCCGGGTATTAGTAACACTGTGCCAAGCG

Table S4. List of primers used for colony PCR to confirm the genotypes of WT and Δ*ythA B. megaterium* strains.

SI References

1. J. A. Birrell, G. Yakovlev, J. Hirst, Reactions of the Flavin Mononucleotide in Complex I: A Combined Mechanism Describes NADH Oxidation Coupled to the Reduction of $APAD^+$, Ferricyanide, or Molecular Oxygen. *Biochemistry* **48**, 12005–12013 (2009).
